# Sensitization of Gram-Negative Bacteria to Aminoglycosides with 2-Aminoimidazole Adjuvants

**DOI:** 10.3390/antibiotics12111563

**Published:** 2023-10-25

**Authors:** Ashley N. Crotteau, Veronica B. Hubble, Santiana A. Marrujo, Anne E. Mattingly, Roberta J. Melander, Christian Melander

**Affiliations:** Department of Chemistry and Biochemistry, University of Notre Dame, Notre Dame, IN 46556, USA; acrottea@nd.edu (A.N.C.);

**Keywords:** *Acinetobacter baumannii*, antibiotic resistance, aminoglycosides, antibiotic adjuvant

## Abstract

In 2019, five million deaths associated with antimicrobial resistance were reported by The Centers for Disease Control and Prevention (CDC). *Acinetobacter baumannii*, a Gram-negative bacterial pathogen, is among the list of urgent threats. Previously, we reported 2-aminoimidazole (2-AI) adjuvants that potentiate macrolide activity against *A. baumannii*. In this study, we identify several of these adjuvants that sensitize *A. baumannii* to aminoglycoside antibiotics. Lead compounds **1** and **7** lower the tobramycin (TOB) minimum inhibitory concentration (MIC) against the TOB-resistant strain AB5075 from 128 μg/mL to 2 μg/mL at 30 μM. In addition, the lead compounds lower the TOB MIC against the TOB-susceptible strain AB19606 from 4 μg/mL to 1 μg/mL and 0.5 μg/mL, respectively, at 30 μM and 15 μM. The evolution of resistance to TOB and **1** in AB5075 revealed mutations in genes related to protein synthesis, the survival of bacteria under environmental stressors, bacteriophages, and proteins containing Ig-like domains.

## 1. Introduction

In recent decades, there has been a significant increase in infections caused by multi-drug resistant (MDR) bacteria. In the United States alone, ca. 2.8 million people are infected by MDR bacteria annually, resulting in the loss of more than 35,000 lives [1]. Some of the most problematic MDR bacterial species have been termed the ESKAPE pathogens (*Enterococcus faecium*, *Staphylococcus aureus*, *Klebsiella pneumoniae*, *Acinetobacter baumannii*, *Pseudomonas aeruginosa*, and *Enterobacter* species) for that fact that they evade/escape commonly used antibiotics [2].

Despite the increased incidence of MDR bacterial infections, investment in the development of new antibiotics has slowed considerably over the last few decades [3]. Within the past 60 years, only two novel classes of antibiotics have been introduced to the clinic, the lipopeptides (i.e., daptomycin) and the oxazolidinones (i.e., linezolid), both of which are Gram-positive selective, and resistance to these antimicrobial agents emerged within just a few years of deployment [4,5]. Gram-negative bacteria are inherently more resistant to many antibiotics than Gram-positive bacteria, in part due to the low permeability of the outer membrane (OM), contributing to the failure to identify novel Gram-negative acting antibiotics in recent decades [6].

The aminoglycosides are oligosaccharide-based antibiotics that are commonly employed to treat infections caused by both Gram-positive and Gram-negative bacteria [7]. Resistance to aminoglycosides is increasingly prevalent and occurs through multiple mechanisms, including enzymatic drug modification, target site modification, and changes in cell permeability [7]. In addition to aminoglycoside resistance, patient toxicity is a cause for concern. Ototoxicity is observed in about 10% of patients receiving aminoglycosides intravenously [8], while nephrotoxicity is seen in 10–25% of therapeutic courses [9]. In order to improve and extend aminoglycoside therapy, both resistance and toxicity need to be addressed. One approach to overcoming resistance is to pair aminoglycosides with other antibiotics, specifically β-lactams, which act synergistically to enhance aminoglycoside uptake [10].

Another approach to overcome bacterial resistance that has been employed by us and others is the use of antibiotic adjuvants [11]. Adjuvants are compounds that typically have little to no inherent antibacterial activity, but instead enhance the activity of an antibiotic. Adjuvants can be employed to render resistant bacterial strains susceptible to an antibiotic [12], or to further enhance activity against a susceptible strain, thereby enabling lower antibiotic dosing which has the potential to reduce dose-dependent toxicity [13,14,15]. To identify new classes of adjuvants, our group has investigated analogs of sponge-derived marine alkaloids containing a 2-aminoimidazole (2-AI) heterocycle and has demonstrated that properly derivatized 2-AIs potentiate a variety of antibiotics against a multitude of MDR and susceptible bacteria. For example, we have demonstrated that specific 2-AI analogs enhance the efficacy of colistin, the antibiotic of last resort used to treat MDR Gram-negative infections, against both resistant and susceptible strains [16,17]. Recently, we have shown that aryl 2-AIs and dimeric 2-AIs potentiate the Gram-positive selective macrolide class of antibiotics against the Gram-negative bacterium *A. baumannii* [18,19,20].

Preliminary mechanism of action studies led us to hypothesize that macrolide potentiation in *A. baumannii* occurs as a result of altered lipooligosaccharide (LOS) biosynthesis/presentation [19], which leads to an increase in membrane permeability. As molecules that increase membrane permeability through direct binding act as adjuvants for a wide variety of antibiotic classes [21,22,23], we investigated the possibility that aryl-2-AI adjuvants potentiate aminoglycoside activity against *A. baumannii* and potentially other Gram-negative pathogens. Herein, we report that aryl 2-AI adjuvants reverse aminoglycoside resistance in aminoglycoside-resistant *A. baumannii,* and *K. pneumoniae*. In addition, we show that these compounds further sensitize aminoglycoside-susceptible strains to the effects of aminoglycosides. These results highlight the potential use of adjuvants to both overcome aminoglycoside resistance and mitigate aminoglycoside toxicity.

## 2. Results

### 2.1. Initial Screen

**Figure 1:**
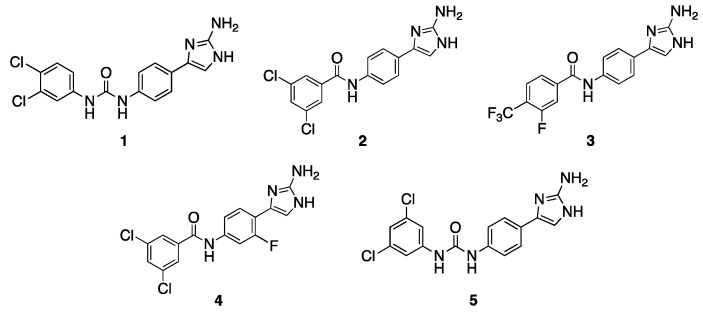
Initial compounds screened for potentiation of TOB.

We began this study by evaluating five 2-AI-aryl analogs (Figure 1) that potentiate clarithromycin against *A. baumannii* [18,19] for their effect upon tobramycin (TOB) activity against *A. baumannii* strain 5075 (AB5075, tobramycin resistant) and *A. baumannii* strain 19606 (AB19606, tobramycin susceptible). Initially, we determined the MIC of each compound individually (Table S1). Compound **4** [18] is the least toxic, returning an MIC of >200 μM against both strains. Compounds **1** [18] and **2** [24] registered MICs of 100 μM against both strains, while compounds **3** [24] and **5** [18] returned MICs ranging from 25–100 μM. Next, the MIC of TOB was determined in the presence and absence of adjuvant at 30% MIC (Table 1). The activity was defined based upon the Clinical and Laboratory Standards Institute (CLSI) breakpoint for TOB against *A. baumannii* (4 μg/mL) [25] in AB5075, or by a minimum of four-fold reduction in the TOB MIC in AB19606.

Compound **1**, which contains a urea linker, para-2-AI, and 3,4-dichlorophenyl tail, lowers the TOB MIC from 128 μg/mL to 2 μg/mL (64-fold) against AB5075, and from 4 μg/mL to 1 μg/mL (four-fold) against AB19606, both at 30 μM. Similar to **1**, compounds **2** and **3**, which both contain an amide linker, para-2-AI, and a di-substituted aryl tail, also lower the TOB MIC in AB19606, returning MICs of 1 μg/mL (four-fold) and 0.5 μg/mL (eight-fold), respectively. However, these compounds do not lower the TOB MIC to the breakpoint in AB5075. Compound **4**, which is similar in structure to compound **2**, differing only by the addition of a meta-fluoro substituent on the phenyl core, reduces the TOB MIC four-fold and two-fold against AB5075 and AB19606, respectively, at 60 μM. Compound **5**, which contains a urea linker, para-2-AI, and 3,5-dichlorophenyl tail, reduces the TOB MIC by the same magnitude as compound **4** against both strains but was tested at a much lower concentration (7.5 μM) due to its higher standalone toxicity.

### 2.2. Follow-Up Screen of Additional Analogues

**Figure 2:**
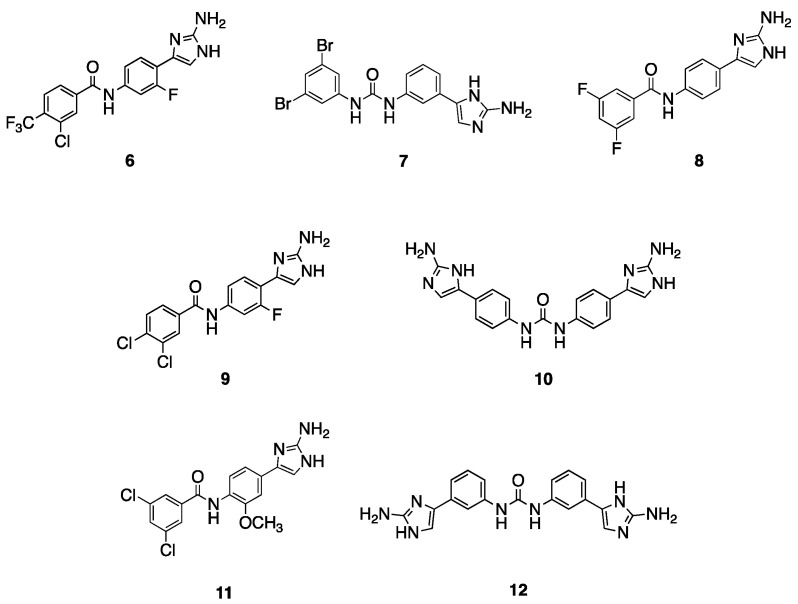
Active compounds from follow-up screen.

Based upon this initial screen, an additional 41 related analogs from our in-house library were screened for potentiation of TOB (Figure S1), leading to the identification of several compounds with adjuvant activity against both AB5075 and AB19606 (Table 2 and Table S3), the seven most active compounds of which are depicted in Figure 2 (**6**–**12**). Compounds **6** [18] and **7** [26] lower the TOB MIC against both the resistant and susceptible *A. baumannii* strains. Compound **6**, which has an amide linker, para 2-AI, and a 3-chloro-4-trifluoromethylphenyl tail, lowers the TOB MIC from 128 μg/mL to 4 μg/mL (32-fold) against AB5075 and from 4 μg/mL to 0.5 μg/mL (8-fold) against AB19606, both at 30 μM. Compound **7**, which contains a urea linker, meta-2-AI, and a 3,5-dibromophenyl tail, potentiates TOB to the same degree as compound **6** against AB19606, but at a lower concentration (15 μM), and similar activity against AB5075 reducing the TOB MIC from 128 μg/mL to 2 μg/mL (64-fold). Compound **8** [27], which has an amide linker, para-2-AI, and 3,5-difluorophenyl tail, has an identical activity to **6** against AB5075 at 60 μM, however, is inactive against AB19606. Similar in structure to **6**, compound **9** [18], which contains an amide linker, the meta-fluoro substituent on the phenyl core, and a 3,4-disubstitutedphenyl tail, lowers the TOB MIC from 4 μg/mL to 0.0625 μg/mL (64-fold) against AB19606. Compound **10** [20], which is a symmetrical para-2-AI dimer, lowers the TOB MIC from 4 μg/mL to 0.25 μg/mL against AB19606. Both **9** and **10** exhibit minimal activity (4- to 8-fold reduction in MIC) against AB5075. The methoxy substituted 3,5-dichloro tail analogue **11** [18] reduces the TOB MIC 8-fold against AB5075, and 4-fold against AB19606. Finally, the meta-meta linked dimer **12** [20] lowers the TOB MIC 16-fold against AB5075 and 8-fold against AB19606.

### 2.3. Activity of Lead Compounds with Additional Aminoglycosides

We next evaluated whether lead compounds sensitize *A. baumannii* to the additional aminoglycosides: streptomycin (STM), kanamycin (KAN), neomycin (NEO), and gentamicin (GEN). Compounds **1**, **6**, **7**, and **8** were chosen for evaluation against AB5075, and compounds **1**, **3**, **5**, **6**, **7**, and **10** were chosen for evaluation against AB19606. AB5075 is resistant to all aminoglycosides tested, while AB19606 is susceptible to kanamycin and neomycin, but resistant to streptomycin and gentamicin. Compounds lowered the STM MIC ranging from 4- to 64-fold in AB5075 (Table 3) and 0- to 32-fold in AB19606 (Table 4). Compound **6** is the most active potentiator of STM of the compounds tested, reducing the MIC from 2048 μg/mL to 32 μg/mL (64-fold) in AB5075 and 512 μg/mL to 16 μg/mL (32-fold) in AB19606, both at 30 μM.

We next assessed KAN potentiation in AB5075 and noted that none of the compounds lowered the KAN MIC more than 2-fold, registering MICs no lower than 1024 μg/mL (from 2048 μg/mL). Four compounds, however, do lower the KAN MIC to a range of 0.5–2 μg/mL (from 8 μg/mL) in AB19606 (Table 5). Against AB19606, compound **10** is the most active, eliciting a 16-fold reduction of the KAN MIC at a concentration of 60 μM. Compounds **6** and **7** are also active, returning KAN MICs of 1 μg/mL at concentrations of 30 and 15 μM, respectively.

The third aminoglycoside evaluated was NEO, and we observed that compound **8** has the highest activity against AB5075, lowering the MIC by 16-fold, from 64 μg/mL to 4 μg/mL. All other compounds are markedly less active, and only potentiate NEO against AB5075 by no more than 4-fold. In AB19606, the compounds active with TOB also showed adjuvant activity with NEO. All compounds, with the exception of compound **1**, lower the NEO MIC by 4- to 16-fold (from 8 μg/mL), with compound **10** being the most active, lowering the NEO MIC to 0.5 μg/mL at 60 μM.

Finally, we evaluated compounds for potentiation of GEN. Interestingly, against AB19606 these compounds exhibit the most potent activity in combination with GEN when compared to the other aminoglycosides. All compounds lower the GEN MIC by at least 4-fold, with only one compound (**6**) not lowering the MIC to the GEN breakpoint (4 μg/mL) or below. Compound **10** is the most potent, lowering the MIC by 64-fold to 0.25 μg/mL in AB19606. In AB5075, however, no compounds lower the GEN MIC to the breakpoint. The largest drop in GEN MIC is affected by compound **8,** which lowers the MIC to 16 μg/mL from 512 μg/mL in AB5075.

### 2.4. Activity of Lead Compounds against Other Gram-Negative Pathogens

Next, we probed the spectrum of activity of lead adjuvants **1**, **3**, **5**, **10**, **11**, and **12** against two additional Gram-negative species *K. pneumoniae* and *E. coli.* A TOB-resistant and a TOB-sensitive strain of *K. pneumoniae* (KP1705/KP43816), and a TOB-resistant and a TOB-sensitive strain of *E. coli* (EC197/EC199) were assayed (Table 5). Compound **12** shows a modest 4-fold reduction in TOB MIC against the TOB-resistant strain KP1705 at 60 μM, while compounds **1**, **5,** and **10** lower the TOB MIC against the TOB-susceptible strain KP43816 at concentrations of 15 μM, 15 μM, and 30 μM, respectively. No TOB adjuvant activity was observed against either of the *E. coli* strains, and no activity was seen for compound **11** against any strain.

### 2.5. Effect of Divalent Cations on Adjuvant Activity

We probed if the TOB adjuvant activity of the lead compounds would be affected by the stabilization of the OM with the addition of MgCl_2_ (Table S5). Supplementation with MgCl_2_ (20 mM) led to a loss or significant decrease in activity. In AB5075, the TOB adjuvant activity dropped from a 64-fold reduction to only a 4-fold reduction in activity. Similarly, in AB19606, the activity went from an 8-fold reduction to a 2-fold reduction with the addition of MgCl_2_.

### 2.6. Evolution of TOB Resistance in the Presence and Absence of Adjuvant and Identification of Mutated Genes in Evolved Strains

To gauge the effect of adjuvants on resistance acquisition, an evolution experiment was carried out by serially passaging AB5075 in the presence of TOB, at the MIC and ± 2-fold the MIC, both in the presence and absence of 15 μM adjuvant **1**. MICs were recorded every other day for eight days (Table 6). Unsurprisingly, resistance in the bacterial population serially passaged in TOB only increased, with an MIC of 4096 μg/mL (32-fold) recorded after passaging for six days. TOB resistance in the population of AB5075 passaged in the presence of TOB and **1** also increased, with a 16-fold increase to 2048 μg/mL on day eight. No major differences were seen in the evolution of TOB resistance when serially passaged in TOB only or in TOB + **1** (Table 6). Compound **1**, at 15 μM, does not lower the TOB MIC against the strain evolved in the presence of TOB only, returning an MIC of 2048 μg/mL (Table S6). In addition, TOB potentiation, in the presence of compound **1** at 15 μM against AB5075 serially passaged in TOB + **1**, was lost by day four, showing only a 2-fold change in MIC in the presence and absence of 15 μM compound **1** (Table S7).

Whole genome sequencing revealed mutations in ten shared genes between the two evolved strains (Table 7). Eleven mutations were observed in alkyl hydroperoxide reductase subunit F (*ahpF*). Mutations were also observed in genes encoding: an aldehyde dehydrogenase, a bacteriophage protein, a stress-induced protein, a replication initiation factor domain-containing protein, an Ig-like domain repeat protein, and four hypothetical proteins. One of the hypothetical proteins is homologous with an elongation factor Tu (*tuf*), while another has 98% homology with a replication protein previously found in *A. baumannii* [28]. No homology was detected for the remaining two hypothetical proteins via NCBI Blast.

## 3. Discussion

Our initial screen revealed that in addition to acting as a macrolide adjuvant, compound **1** also potentiates the aminoglycoside TOB against the resistant strain AB5075 (64-fold reduction in MIC), in addition to moderately increasing activity against the susceptible strain AB19606 (4-fold reduction in MIC). A follow-up screen identified several additional active compounds that sensitize both resistant and susceptible *A. baumannii* to TOB, the most active of which are compound **7** (64-fold reduction against AB5075), and compound **9** (64-fold reduction against AB19606).

Lead compounds do not lower the MIC of any additional aminoglycosides to the breakpoint in AB5075, but do potentiate all the additional aminoglycosides examined, with the exception of STM against AB19606 (to which AB19606 is highly resistant). Lead compounds also do not potentiate TOB against *E. coli*, and exhibit very little TOB potentiation against *K. pneumoniae*, suggesting a selectivity for TOB, and for *A. baumannii*. Comparatively, Yarlagadda et al. reported that the natural product venturicidin A potentiates GEN 4- to 8-fold against some strains of *A. baumannii* and *P. aeruginosa*, but not *E. coli* or *K. pneumoniae,* and was posited to act via inhibition of ATP synthesis [29]. Hui et al. reported moderate enhancement (4-fold reduction in MIC) of KAN and GEN activity against *A. baumannii* and *K. pneumoniae* by a benzoisothiazolone that was shown to interact with the peroxidase OhrB from *A. baumannii* 19606 [30].

Mg^2+^ ions stabilize the OM through electrostatic interactions [31], and known OM disrupters, such as pentamidine and PMBN, do not potentiate rifampicin in the presence of Mg^2+^ ions [22]. Upon supplementation of the media with MgCl_2_, adjuvant activity is essentially eradicated, which could be due to inhibition of uptake of the adjuvant, or to direct blocking of adjuvant activity. Marrujo et al. have previously shown that the addition of exogenous LOS from AB5075 resulted in no loss in macrolide potentiation activity of the class of dimeric 2-AI adjuvants to which compound **10** belongs [20], suggesting that the 2-AI adjuvants do not directly bind the membrane. Further mechanistic studies to determine if adjuvants are affecting biosynthesis or assembly of LOS are ongoing.

Unlike previous resistance evolution studies with related adjuvants and antibiotic classes [17,18,32], the serial passage in TOB, in both the absence and presence of **1**, leads to dramatic increases in the TOB MIC of 32-fold and 16-fold, respectively. Adjuvant **1** does not potentiate TOB against either evolved strain. Exploration of shared gene mutations between bacterial populations evolved in the presence of either TOB alone or TOB and adjuvant **1** revealed that by far the highest number of mutations occurred in the alkylhydroperoxidase subunit F gene (*ahpF*), which encodes a subunit of the ROS detoxification enzyme AhpCF [33]. It has been reported by Dwyer et al. that AhpCF overexpression confers some protection from the oxidative stress-induced lethality of bactericidal antibiotics including GEN [34], while Lv et al. showed that overexpression enhances resistance to heat-shock-induced TOB lethality [35] in *E. coli.* The latter report also showed that heat shock similarly potentiates TOB against *A. baumannii,* although the mechanistic basis of this phenomenon in *A. baumannii* was not explored [35]. Aldehyde dehydrogenase plays a role in a variety of metabolic processes, including redox regulation and environmental stress defense, and has been reported to be differentially expressed between MDR and non-MDR *A. baumannii* isolates [36]. It is not surprising that mutations that impact the ability of the bacterium to respond to ROS and other stressors lead to reduced susceptibility to TOB; however, the effects of the mutations on protein function remain to be determined.

## 4. Materials and Methods

### 4.1. Bacterial Strains, Media, and Antimicrobial Agents

*A. baumannii* strain 5075 was obtained from Walter Reed Army Institute for Research (WRAIR). *A. baumannii* strain 19606, *K. pneumoniae* strains BAA-1705, and 43816, and *E. coli* strains BAA-197, and BAA-199 were obtained from American Type Culture Collection (ATCC). Stock cultures of these strains were stored in 25% glycerol and maintained at −80 °C. Prior to use colonies were grown on LB (Lennox) agar. Cation-adjusted Mueller–Hinton II Broth (CAMHB) (cat# 21322) was purchased from BD Diagnostics (Franklin Lakes, NJ, USA). LB broth (cat# BP1427-2) was purchased from Fisher Scientific (Waltham, MA, USA). Tobramycin (cat# T2503) and gentamicin (cat# G0383) were purchased from TCI. Streptomycin (cat# S9137), kanamycin (cat# K1377), and neomycin (cat# N6386) were purchased from Sigma Life Science (Burlington, VT, USA). All assays were completed in duplicate and repeated at least two separate times. Syntheses of compounds have been previously reported, and compounds were dissolved as their HCl salts in molecular biology grade DMSO as 5, 10, or 100 mM stock solutions and stored at −20 °C. Aminoglycosides were dissolved in sterile water.

### 4.2. Broth Microdilution Method for Minimum Inhibitory Concentration Determination

Day cultures (4–6 h) in CAMHB were subcultured to 5 × 10^5^ CFU/mL in fresh CAMHB. Aliquots (1 mL) were placed in culture tubes and compound was added from 100 mM stock samples in DMSO, such that the compound concentration equaled highest concentration tested (200 μM). Then 200 μL of sample were dispensed into the first row of a 96-well microtiter plate, while the remaining rows, except the final row, were filled with 100 μL of the initial bacterial subculture. The final row was filled with CAMHB to serve as a sterility control. Row 1 wells were mixed five times, and then 100 μL was transferred to the second row. The second row was then mixed five times and 100 μL was transferred to row 3. This process was repeated until all rows, except the final row had been mixed. Plates were covered with GLAD Press n’ Seal and incubated under stationary conditions at 37 °C for 16–18 h. The OD_600_ of each well was recorded using a BioTek Synergy HTX multi-mode reader plate reader from Agilent Technologies (Santa Clara, CA, USA). MIC values were determined as the minimum concentration required to achieve 90% bacterial growth inhibition compared to growth in untreated wells [37].

### 4.3. Broth Microdilution Method for Measurement of Aminoglycoside Potentiation

Day cultures (6 h) of each bacterial strain in CAMHB were subcultured to 5 × 10^5^ CFU/mL in CAMHB. Aliquots (4 mL) were placed in culture tubes and the compound was dosed from 5, 10, or 100 mM stock samples to give the desired concentration of the compounds to be tested. An amount of 1 mL of the resulting solution was aliquoted into a separate culture tube and dosed with antibiotics at the highest concentration to be tested. Bacteria treated with antibiotics alone served as the control. Row 1 of a 96-well plate was filled with 200 μL of the antibiotic/compound aliquot, and rows 2–11 were filled with 100 μL each of the remaining 4 mL bacterial subcultures containing adjuvant at the desired concentration. Row 12 was filled with CAMHB to serve as a sterility control. Row 1 was mixed five times and 100 μL was transferred to row 2, which was then mixed five times and transferred to row 3. This process was repeated until all rows had been mixed, except for row 11 which had only compound to serve as a control to monitor toxicity. Plates were covered with GLAD Press n’ Seal and incubated under stationary conditions at 37 °C for 16–18 h. The OD_600_ of each well was recorded. MIC values were determined as the minimum concentration required to achieve 90% bacterial growth inhibition compared to growth in untreated wells.

### 4.4. Evolution of Tobramycin Resistance in A. baumannii

*A. baumannii* strain 5075 was grown in CAMHB overnight and subcultured to 5 x 10^5^ CFU/mL in 6 mL of fresh CAMHB. Adjuvant **1** was dosed at 15 μM from a DMSO stock solution. Aliquots (1 mL) were placed into five separate culture tubes and tobramycin was dosed at the desired concentrations from a stock solution and incubated with shaking at 37 °C overnight. Conditions were as follows: 2× and 4× above the active tobramycin MIC (no visible bacterial growth) with or without 15 μM adjuvant **1**, 2× and 4× below the active tobramycin MIC with or without 15 μM adjuvant **1**, and tobramycin at the active MIC with or without 15 μM adjuvant **1**. After 24 h bacterial growth was evaluated, and the sample with the highest tobramycin concentration where turbidity was observed was subcultured to 5 × 10^5^ CFU/mL and incubated with shaking overnight. This process was repeated for eight days with MICs determined by broth microdilution as described above every two days (Table S5). For whole genome sequencing, the day eight evolved AB5075 cultures were plated on LB agar and let grow overnight. A single colony was picked and grown overnight in CAMHB. The overnight culture was centrifuged at 5000 rpm (1700 g) for three minutes. The pellets were washed with 0.5 mL of sterile water and centrifuged at 5000 rpm for three minutes. The supernatant was discarded, and the pellets were resuspended in 200 μL DNA suspension buffer (0.1 mM EDTA, 10 mM Tris HCl). Whole genome sequencing and analysis (Supplementary Excel) of the AB5075 evolved strains, as well as the parent, were carried out by CosmosID. 

### 4.5. Sequencing Data Analysis

CosmosID provided an Excel document with a list of genetic differences between the parent and evolved AB5075 strains and an annotated AB5075 reference (gabab5075v1.0: https://www.ncbi.nlm.nih.gov/assembly/GCF_000770605.1, accessed on 17 October 2022). Further analysis was completed using the provided list where the differences between wild-type AB5075 and the reference strain were compared to the differences between evolved AB5075 strains and the reference strain. A list of genetic differences between the parent AB5075 and evolved strains was compiled. Hypothetical proteins were analyzed using NCBI Blast.

## 5. Conclusions

In conclusion, we report 2-AI analogs that sensitize MDR and susceptible strains of Gram-negative bacteria to aminoglycoside antibiotics. Compounds **1** and **7** are the most active adjuvants against both the resistant and susceptible strains of *A. baumannii*, enhancing TOB activity by 64-fold against the MDR strain, AB5075, and up to 8-fold against the TOB susceptible strain AB19606. Compound **9** is the most active adjuvant against the TOB susceptible, AB19606, increasing the TOB MIC by 64-fold. Compound **10** is the most effective adjuvant with other aminoglycosides including, NEO, GEN, and KAN against AB19606. In addition, compound **10** exhibits the most potent activity with TOB against *K. pneumoniae*. Finally, a resistance evolution experiment revealed ten genes including *ahpF,* which encodes an alkylhydroperoxidase subunit, that may play a role in aminoglycoside resistance.

## Figures and Tables

**Table 1:** TOB MICs in the presence of compounds **1**–**5** against AB5075 and AB19606.

	AB5075		AB19606	
Compound	Concentration (μM)	TOB MIC (μg/mL) (Fold-Reduction)	Concentration (μM)	TOB MIC (μg/mL) (Fold-Reduction)
		128		4
**1**	30	2 (64)	30	1 (4)
**2**	30	16 (8)	30	1 (4)
**3**	15	64 (2)	30	0.5 (8)
**4**	60	32 (4)	60	2 (2)
**5**	7.5	32 (4)	15	2 (2)

**Table 2:** TOB MICs in the presence of compounds **6**–**12** against AB5075 and AB19606.

	AB5075		AB19606	
Compound	Concentration (μM)	TOB MIC (μg/mL) (Fold-Reduction)	Concentration (μM)	TOB MIC (μg/mL) (Fold-Reduction)
		128		4
**6**	30	4 (32)	30	0.5 (8)
**7**	30	2 (64)	15	0.5 (8)
**8**	60	4 (32)	60	2 (2)
**9**	60	16 (8)	60	0.0625 (64)
**10**	30	32 (4)	60	0.25 (16)
**11**	60	16 (8)	30	1 (4)
**12**	60	8 (16)	60	0.5 (8)

**Table 3:** Additional aminoglycoside MICs in the presence of select 2-AI compounds against AB5075.

Compound	Concentration (μM)	STM MIC (μg/mL) (Fold-Reduction)	Concentration (μM)	KAN MIC (μg/mL) (Fold-Reduction)	Concentration (μM)	NEO MIC (μg/mL) (Fold-Reduction)	Concentration (μM)	GEN MIC (μg/mL) (Fold-Reduction)
		2048		2048		64		512
**1**	30	128 (16)	15	2048 (0)	15	32 (2)	15	32 (16)
**6**	30	32 (64)	30	1024 (2)	30	16 (4)	30	>512 (0)
**7**	15	128 (16)	7.5	2048 (0)	7.5	>64 (0)	7.5	256 (2)
**8**	30	512 (4)	60	2048 (0)	60	4 (16)	60	16 (32)

STM = Streptomycin, KAN = Kanamycin, NEO = Neomycin, GEN = Gentamicin.

**Table 4:** Additional aminoglycoside MICs in the presence of select 2-AI compounds against AB19606.

Compound	Concentration (μM)	STM MIC (μg/mL) (Fold-Reduction)	Concentration (μM)	KAN MIC (μg/mL) (Fold-Reduction)	Concentration (μM)	NEO MIC (μg/mL) (Fold-Reduction)	Concentration (μM)	GEN MIC (μg/mL) (Fold-Reduction)
		512		8		8		32
**1**	30	128 (4)	15	>8 (0)	15	4 (2)	15	4 (8)
**3**	30	64 (8)	30	2 (4)	30	1 (8)	30	1 (32)
**5**	15	64 (8)	15	4 (2)	15	2 (4)	15	2 (16)
**6**	30	16 (32)	30	1 (8)	30	1 (8)	30	32 (0)
**7**	7.5	512 (0)	15	1 (8)	15	2 (4)	15	4 (8)
**10**	60	16 (32)	60	0.5 (16)	60	0.5 (16)	60	0.25 (128)
					30	2 (4)	45	2 (16)

STM = Streptomycin, KAN = Kanamycin, NEO = Neomycin, GEN = Gentamicin.

**Table 5:** Tobramycin MICs against *K. pneumoniae* and *Escherichia coli*.

Strain	TOB MIC (μg/mL)	TOB MIC (μg/mL) + 1 (μM)	TOB MIC (μg/mL) + 3 (μM)	TOB MIC (μg/mL) + 5 (μM)	TOB MIC (μg/mL) + 10 (μM)	TOB MIC (μg/mL) + 11 (μM)	TOB MIC (μg/mL) + 12 (μM)
KP1705	32	(15) 16	(15) 32	(15) 16	(60) 16	(60) 32	(60) 8
KP43816	1	(15) 0.125	(15) 0.5	(15) 0.125	(30) 0.25	(60) 0.5	(10) 0.5
EC197	32	(3.75) 32	(7.5) 16	(7.5) 16	(15) 16	(60) 16	(15) 16
EC199	0.5	(7.5) 0.5	(7.5) 0.5	(7.5) 0.5	(15) 0.5	(60) 0.5	(15) 0.5

Concentration of adjuvant (μM) indicated in parentheses.

**Table 6:** Tobramycin MICs (μg/mL) of serially passaged bacterial populations.

	AB5075 Passaged in TOB Only	AB5075 Passaged in TOB + 1 (15 μM)
Day 0	128	128
Day 2	1024	256
Day 4	1024	1024
Day 6	4096	1024
Day 8	4096	2048

**Table 7:** Shared genes with mutations following serial passage.

Gene Product (*Gene*)	Locus Tag	# of Mutations	Type of Mutation
Alkyl hydroperoxide reductase subunit F (*ahpF*)	A591_RS08360	11	8 snp; 3 complex
Aldehyde dehydrogenase	A591_RS16100	3	1 snp; 2 complex
Bacteriophage protein	A591_RS13885	3	2 snp; 1 complex
Stress-induced protein	A591_RS20205	1	snp
Replication initiation factor domain-containing protein	A591_RS15850	1	Insertion
Ig-like domain repeat protein	A591_RS19590	1	snp
Hypothetical protein (elongation factor Tu (*tuf*))	A591_RS01280	1	snp
Hypothetical protein (replication protein)	A591_RS20250	1	Insertion
Unknown hypothetical protein	A591_RS20245	1	Insertion
Unknown hypothetical protein	A591_RS15845	1	Insertion

## Data Availability

All data are provided in the manuscript and supplemental information.

## Supplementary Materials

The following supporting information can be downloaded at: https://www.mdpi.com/article/10.3390/antibiotics12111563/s1, Table S1: Minimum inhibitory concentration data of initial compounds tested against AB5075 and AB19606; Figure S1: Structures of additional analogs tested in combination with TOB against *A. baumannii*; Table S2: Additional compound MICs against AB5075 and AB19606; Table S3: TOB MICs in combination with all additional analogs against AB5075 and AB19606; Table S4: Compound MICs against a panel of *K. pneumoniae* and *E. coli* strains; Table S5: TOB MICs in the presence of 20 mM MgCl_2_; Table S6: Tobramycin MICs in (TOB only) evolved AB5075; Table S7: TOB potentiation in the presence and absence of compound 1 in AB5075 serially passaged in TOB +1; Supplemental Excel: Tab S1: Parent AB5075; Tab S2: TOB evolved AB5075; Tab S3: TOB + 1 evolved AB5075; Tab S4: Same gene differences (Evolved).
