# Supplementary material for: Sensitization of Gram-Negative Bacteria to Aminoglycosides with 2-Aminoimidazole Adjuvants
Source: Antibiotics (Basel). 2023 Oct 25;12(11):1563. doi: 10.3390/antibiotics12111563 (PMC10668796; doi:10.3390/antibiotics12111563)
Supplement: Supplementary file 1 [file antibiotics-12-01563-s001.zip › Aminoglycoside Paper Supplementary Information.pdf]

Sensitization of Gram-Negative Bacteria to Aminoglycosides with 2-aminoimidazole Adjuvants

Ashley N. Crotteau, Veronica B. Hubble, Santiana A. Marrujo, Anne E. Mattingly, Roberta J. Melander, and Christian Melander*

Department of Chemistry and Biochemistry, University of Notre Dame, Notre Dame, IN 46556, USA; acrottea@nd.edu (A.C.)

*Correspondence: cmelande@nd.edu

Table of Contents

Biological Data 2
Table S1. Minimum inhibitory concentration data of initial compounds tested against AB5075 and AB19606. 2
Figure S1. Structures of additional analogs tested in combination with TOB against *A. baumannii*. 2
Table S2. Additional compound MICs against AB5075 and AB19606. 3
Table S3. TOB MICs in combination with all additional analogs against AB5075 and AB19606. 5
Table S4. Compound MICs against a panel of *K. pneumoniae* and *E. coli* strains. 6
Table S5. TOB MICs in the presence of 20 mM $MgCl_2$. 6
Table S6. Tobramycin MICs in (TOB only) evolved AB5075. 6
Table S7. TOB potentiation in the presence and absence of compound 1 in AB5075 serially passaged in TOB + 1. 7

Biological Data

Table S1. Minimum inhibitory concentration data of initial compounds tested against AB5075 and AB19606.

Compound	MIC (μM) against AB5075	MIC (μM) against AB19606
**1**	100	100
**2**	100	100
**3**	50	100
**4**	>200	>200
**5**	25	50

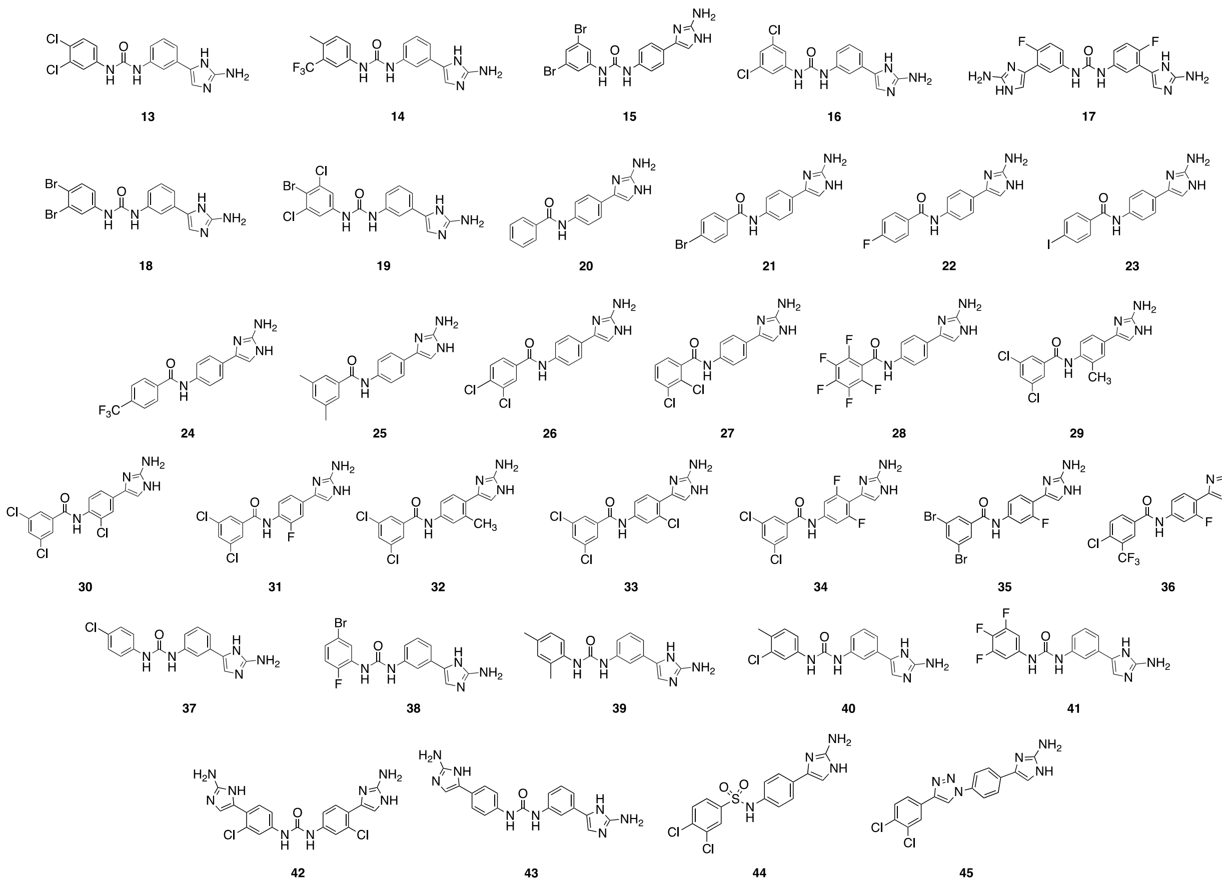

Figure S1. Structures of additional analogs tested in combination with TOB against *A. baumannii*.

Table S2. Additional compound MICs against AB5075 and AB19606.

Compound	MIC (μM) against AB5075	MIC (μM) against AB19606
**6**	200	100
**7**	100	50
**8**	200	200
**9**	100	>200
**10**	100	>200
**11**	200	100
**12**	200	200
**13**	100	100
**14**	>200	>200
**15**	50	50
**16**	200	100
**17**	>200	>200
**18**	100	50
**19**	>200	100
**20**	>200	>200
**21**	>200	>200
**22**	>200	>200
**23**	>200	>200
**24**	>200	>200
**25**	>200	>200
**26**	100	50
**27**	>200	>200
**28**	>200	>200
**39**	>200	>200
**30**	>200	>200
**31**	>200	>200
**32**	>200	>200
**33**	>200	>200
**34**	>200	>200
**35**	>200	>200
**36**	50	50
**37**	>200	>200
**38**	200	100
**39**	>200	>200
**40**	>200	>200
**41**	100	200

**42**	>200	>200
**43**	>200	>200
**44**	>200	>200
**45**	>200	>200

Table S3. TOB MICs in combination with all additional analogs against AB5075 and AB19606.

	AB5075		AB19606	
**Compound**	**Concentration tested (μM)**	**TOB MIC (μg/mL) [fold-reduction]**	**Concentration tested (μM)**	**TOB MIC (μg/mL) [fold-reduction]**
		128		4
**13**	30	16 [8]	30	1 [4]
**14**	60	16 [8]	60	2 [2]
**15**	15	8 [16]	15	2 [2]
**16**	60	8 [16]	30	1 [4]
**17**	10	128 [0]	10	2 [2]
**18**	30	32 [4]	15	>4 [0]
**19**	60	8 [16]	30	0.5 [8]
**20**	60	128 [0]	60	>4 [0]
**21**	60	64 [2]	60	2 [2]
**22**	60	32 [4]	60	4 [0]
**23**	60	128 [0]	60	>4 [0]
**24**	60	128 [0]	60	>4 [0]
**25**	60	128 [0]	60	4 [0]
**26**	30	64 [2]	15	2 [2]
**27**	60	64 [2]	60	>4 [0]
**28**	60	128 [0]	60	>4 [0]
**29**	60	64 [2]	60	2 [2]
**30**	60	128 [0]	60	>4 [0]
**31**	60	128 [0]	60	4 [0]
**32**	60	64 [2]	60	1 [4]
**33**	60	32 [4]	60	1 [4]
**34**	60	128 [0]	60	4 [0]
**35**	60	32 [4]	60	2 [2]
**36**	15	128 [0]	15	2 [2]
**37**	60	64 [2]	60	4 [0]
**38**	60	32 [4]	30	4 [0]
**39**	60	128 [0]	60	>4 [0]
**40**	60	64 [2]	60	>4 [0]
**41**	30	32 [4]	60	2 [2]
**42**	60	128 [0]	60	>4 [0]
**43**	60	64 [2]	60	2 [2]
**44**	60	128 [0]	60	4 [0]
**45**	60	128 [0]	60	4 [0]

Table S4. Compound MICs against a panel of *K. pneumoniae* and *E. coli* strains.

Strain	MIC of 1 (μM)	MIC of 3 (μM)	MIC of 5 (μM)	MIC of 10 (μM)	MIC of 11 (μM)	MIC of 12 (μM)
KP1705	50	50	50	200	>200	>200
KP43816	50	50	50	100	>200	*n.d.
EC197	12.5	25	25	50	200	50
EC199	25	25	25	50	>200	50

*n.d. = not determined

Table S5. TOB MICs in the presence of 20 mM $MgCl_2$.

	AB5075			AB19606		
**Compound**	**Concentration tested (μM)**	**TOB MIC (μg/mL)**	**TOB MIC (μg/mL) + 20 mM $MgCl_2$**	**Concentration tested (μM)**	**TOB MIC (μg/mL)**	**TOB MIC (μg/mL) + 20 mM $MgCl_2$**
		128	512		4	128
**1**	30	2	128			
**3**				30	0.5	64

Table S6. Tobramycin MICs in (TOB only) evolved AB5075.

TOB (μg/mL)	TOB (μg/mL) + 1 (15 μM)
2048	2048

Table S7. TOB potentiation in the presence and absence of compound **1** in AB5075 serially passaged in TOB + **1**.

	**Concentration of compound 1 (µM)**	**TOB MIC (µg/mL)**
Day 0	0	128
	15	16
Day 2	0	256
	15	64
Day 4	0	1024
	15	512
Day 6	0	1024
	15	1024
Day 8	0	2048
	15	2048
